# The contributions of injury deaths to the gender gap in life expectancy and life disparity in Eastern Mediterranean Region

**DOI:** 10.1186/s40621-023-00417-w

**Published:** 2023-01-24

**Authors:** Firoozeh Bairami, Mohammad Hajizadeh, Ali Kiadaliri

**Affiliations:** 1 European Society of Cardiology, Brussels, Belgium; 2 School of Health Administration, Dalhousie University, Halifax, Canada; 3 Clinical Epidemiology Unit, Department of Clinical Sciences Lund, Orthopaedics, Skåne University Hospital, Lund University, Remissgatan 4, 221 85 Lund, Sweden; 4 Centre for Economic Demography, Lund University, Lund, Sweden

**Keywords:** Gender gap, Life expectancy, Life disparity, Avoidable causes of death, Injury death, Eastern Mediterranean Region

## Abstract

**Background:**

Injury deaths constitute a major avoidable cause of death affecting life expectancy to a different degree in men and women. This study quantified the contributions of injury deaths to the gender gap in life expectancy (GGLE) and life disparity (GGLD) in nine Eastern Mediterranean Region (EMR) countries.

**Methods:**

We retrieved annual data on age-sex specific causes of death from the World Health Organization mortality database for EMR countries with at least 2-year consecutive data during 2010–2019. The injury-related deaths were categorized into five groups: transport accidents, other accidental injuries, intentional self-harm, assault and events of undetermined intent. Considering women as the reference, the GGLE and GGLD were decomposed by age and causes of death, using a continuous-change model.

**Results:**

The largest and smallest GGLE were observed in Kuwait (5.2 years) and Qatar (− 1.2 years), respectively. Qatar (− 2.2 years) and Oman (0.2 years) had the highest and lowest GGLD. The highest contributions of injury deaths to the GGLE/GGLD were seen in Libya (1.8/− 1.2 years), followed by Iran (1.2/− 0.8 years). Among injury causes, transport accidents were the leading cause of GGLE in all countries but Libya and Morocco, with Iran having the greatest contributions (0.6 years). Injury deaths in men aged 15–29 years accounted for 33% [41%] (Kuwait) to 55% [65%] (Oman) of total GGLE [GGLD] attributable to injury deaths.

**Conclusions:**

High injury deaths, particularly transport accidents, among young men contributed substantially to the GGLE and GGLD across nine EMR countries in this study. This highlights the need for implementing preventing policies to reduce the burden of injury deaths specifically in young men.

**Supplementary Information:**

The online version contains supplementary material available at 10.1186/s40621-023-00417-w.

## Background

As a major population health measure, life expectancy (LE) is a commonly used indicator to measure a country’s development and health system performance (World Health Organization 2022; Kolip and Lange 2018; Crimmins and Zhang 2019). It reflects the average number of years an individual can expect to live given the current mortality patterns (World Health Organization. Indicators 2022). Although LE provides good information regarding the length of life, it does not demonstrate the variation in length of life in each population. In other words, as LE reflects average, it can mask substantial variation in life span. That is to say, two countries with almost similar LE could experience significant variation in the length of life among their population. Hence, this indicator should be complemented with life disparity (LD) which is a measure that reflects the variation in age at the time of death (Aburto et al. 2020; Raalte et al. 2018). In general, LE can increase by decreasing mortality rate at any age, while reducing premature deaths is necessary to decrease LD. Countries that are working on narrowing the variation in the age at the time of death by reducing premature deaths, are consistently positioned at the top of LE ranking table (Vaupel et al. 2011). A strong positive correlation has been observed between LE at birth and life span equality. The relationship becomes stronger by saving lives at younger ages which translates to higher LE and lower LD (Aburto et al. 2020).

Improvements in living standards and lifestyles, following socioeconomic developments, resulted in a steady increase in LE during the last two centuries (Oeppen and Vaupel 2002). However, this increase was not equal for men and women (Dicker et al. 2018). Even though men are in better position than women in the majority of health indicators particularly in physical functioning measures, women tend to live longer than men in almost all regions and countries (Crimmins et al. 2019). In 2019, global LE for men was reported at 70.9 years, while the corresponding figure for women was 75.9 years (World Health Organization 2021). This persistent gender gap in life expectancy (GGLE) has been attributed to different biological (e.g., genetics and hormones) (Wolff and Gemmell 2013; Pérez-López et al. 2010; Marais et al. 2018) and non-biological factors (e.g., behavioral and social characteristics) (Tan et al. 2019; Rogers et al. 2010). Recent evidence highlights a more significant role of non-biological factors in GGLE (Rogers et al. 2010). Thus, identifying non-biological factors contributing to GGLE and tackling these factors through public health policies have drawn considerable attention in recent years (Allel et al. 2021).

Injuries are a major avoidable cause of death. Deaths caused by injuries contribute to GGLE in favor of women, as men are more vulnerable to severe injuries than women (World Health Organization 2021). Although intentional injuries, for instance suicide attempts, are more recurring in woman, yet men die more often from these injuries (Fox et al. 2018). Unintentional injuries such as road traffic injuries also cause more mortality in men than women (World Health Organization 2021). Injuries impose a considerable burden on the healthcare systems by being the leading cause of death in the younger population and one of the main causes of death in the older population (Abbafati et al. 2020). Injuries including intentional and unintentional stand for 8% of all deaths globally and its contribution to death is more significant in low- and middle-income countries than high-income countries (World Health Organization 2021; World Health Organization 2019). Deaths attributable to injuries in Eastern Mediterranean Region (EMR) countries are threefold higher in low- and middle-income countries compared to high-income countries (World Health Organization 2014). This region in particular stands out by having one of the highest rates of mortality and disability, due to injuries mainly caused by traffic accidents and violence (Mokdad et al. 2018; Khalil et al. 2018). The mortality rate related to injuries in these countries both in the younger and adult population is higher than the global rate (Al-Hajj et al. 2022, 2020). Injury stands for nearly 20% of global child injury deaths in the EMR countries and is the leading cause of death in children and adolescent population of these countries (Al-Hajj et al. 2020). Despite significant contributions of injury-related deaths to the GGLE and GGLD worldwide, and EMR countries in particular, the existing literature on this subject is limited. We aimed to address this knowledge gap by quantifying the contributions of injury-related deaths by age and cause to the GGLE and GGLD across EMR countries using information obtained from the World Health Organization (WHO) mortality database.

## Methods

### Design and setting

We retrieved annual data from the WHO mortality database for the EMR countries. This database includes annual reports based on medically certified deaths from the civil vital registration system (CVRS) of member states. Countries with at least 2-year consecutive data in the mortality database between 2010 and 2019 were included in the study. We used 2-year period for two main reasons: first, most EMR countries don’t report data regularly to the WHO mortality database and hence employing longer period would limit the number of countries eligible, and second, to mitigate the effect of random year-by-year fluctuations. Period 2010–2019 was selected to investigate the most recent period. In this line, we used the most recent 2-year period for each country. To avoid the potential effect of COVID-19 on our estimates, we did not include the data for the year 2020. Our sample included 9 countries from the EMR countries. Four of these countries (Bahrain, Kuwait, Oman and Qatar) are among high-income countries and the remaining five countries (Egypt, Iran, Jordan, Libya, Morocco) are categorized as middle-income countries (World Bank 2022). The following country-year data was available and used in the study: Bahrain 2013–2014, Egypt 2018–2019, Iran 2015–2016, Jordan 2017–2018, Kuwait 2018–2019, Libya 2016–2017, Morocco 2015–2016, Oman 2018–2019 and Qatar 2016–2017. We counted 2-year mortality data. Data were extracted for each country by age group (< 1, 1–4, 5–9, 10–14, …, > 85), sex and underlying cause of death. According to the first five categories under the “external causes of morbidity and mortality” in the 10th revision of International Classification of Diseases (ICD-10), the injury-related deaths were classified into five categories: transport accidents (ICD-10 codes V01-V99), other accidental injuries (W00-X59), intentional self-harm (X60-X84), assault (X85-Y09) and events of undetermined intent (Y10-Y34) (World Health Organization 2019). In this study, we analyzed transport accidents separately from other types of accidents to emphasize the burden of transport accidents. The remaining causes of death were pooled together in a single group as “other causes”.

### Statistical analysis

We used abridged life tables to calculate LE at birth (McCool 1987). To avoid potential country differences in the age and cause specific mortality reports and under-reporting mortality cases in some countries, we adjusted all-cause-age-specific mortality to the WHO life table. We used life disparity (e^†^, “e-dagger”) to measure variation in length of life which quantifies the average remaining life expectancy at death (or alternatively the average years of life lost due to death) (Vaupel and Canudas-Romo 2003; Raalte and Caswell 2013). Life disparity can be expressed as:

$$e^{\dag } = \mathop \smallint \limits_{0}^{m} e\left( x \right){\text{d}}\left( x \right){\text{d}}x$$

where d(*x*) represents the life table distribution of deaths ($\sum {\text{d}}\left( x \right) = 1$), *x* denotes age, *e*(*x*) is life expectancy at age x, and m is the maximum age in the population.

The GGLE and GGLD were decomposed by age and cause of death using continuous-change model suggested by Horiuchi (2008). This is a linear integral decomposition method relying on the assumption that life expectancy/disparity is a differentiable function of the age-cause-specific death rates and effects of these death rates are additive. It also assumes that the age-cause-specific death rates change proportionately along a dimension (e.g. gender) (Horiuchi et al. 2008). Using this method, the difference in life expectancy/disparity between women and men can be written as:

$$y_{w} - y_{m} = \mathop \sum \limits_{i = 1}^{n} \mathop \smallint \limits_{{x_{i(m)} }}^{{x_{i(w)} }} \frac{\partial y}{{\partial x_{i} }}{\text{d}}x_{i} = \mathop \sum \limits_{i = 1}^{n} c_{i}$$

where *y*_*w*_ and *y*_*m*_ represent life expectancy/disparity in women and men, respectively; n represents the number of age groups; xi represents cause-specific mortality for age group i; and c_i_ represents the age-cause-specific contributions to gender gap in life expectancy/disparity. All analyses were conducted in R program using the codes from the following open source: https://github.com/jmaburto.

## Results

### Life expectancy and life disparity

LE at birth was greater for women than men in all countries but Qatar (Table 1). Women in Kuwait (84.8 years) and Morocco (73.6 years) had the highest and the lowest LE, respectively. While the highest LE in men was observed in Kuwait (79.6 years), Egypt (69.6 years) had the lowest LE. The highest and lowest life years lost (life disparity) were observed in Libya and Qatar, respectively, for both sexes. The greatest GGLE were reported in Kuwait (+ 5.2 years), while Qatar had the smallest GGLE (− 1.2 years). The largest and smallest GGLD were reported in Qatar (− 2.2 years) and Oman (+ 0.2 years).

**Table 1:** Gender gap in life expectancy/disparity and contributions of injury deaths across Eastern Mediterranean Region countries

Country	LE, years		GGLE, years		LD, years		GGLD, years	
	Women	Men	Total	Injury death contribution	Women	Men	Total	Injury death contribution
Bahrain	77.3	75.9	1.4	0.5	11.3	11.9	− 0.6	− 0.3
Egypt	74.2	69.6	4.6	0.5	13.6	14.3	− 0.7	− 0.3
Iran	78.7	75.1	3.6	1.2	12.5	14.5	− 2.0	− 0.8
Jordan	79.0	77.3	1.7	0.6	12.3	13.8	− 1.5	− 0.4
Kuwait	84.8	79.6	5.2	0.8	12.4	13.1	− 0.7	− 0.5
Libya	77.2	72.6	4.6	1.8	14.6	16.4	− 1.9	− 1.2
Morocco	73.6	71.4	2.2	0.8	13.6	14.1	− 0.5	− 0.4
Oman	75.3	73.0	2.3	0.4	11.6	11.5	0.2	− 0.3
Qatar	75.9	77.2	− 1.2	0.6	7.8	10.0	− 2.2	− 0.4

LE: life expectancy; LD: Life disparity; GGLE: Gender gap in life expectancy; GGLD: Gender gap in life disparity.

In all countries injury deaths were higher in men than women with contributions of injury deaths to GGLE ranged from 0.4 years in Oman to 1.8 years in Libya. Moreover, since injury deaths were more common among younger age groups, they contributed to greater life years lost in men compared with women ranging from 0.3 years in Oman to 1.2 years in Libya (Table 1).

### Age specific contributions to the GGLE and GGLD

**Fig. 1:**
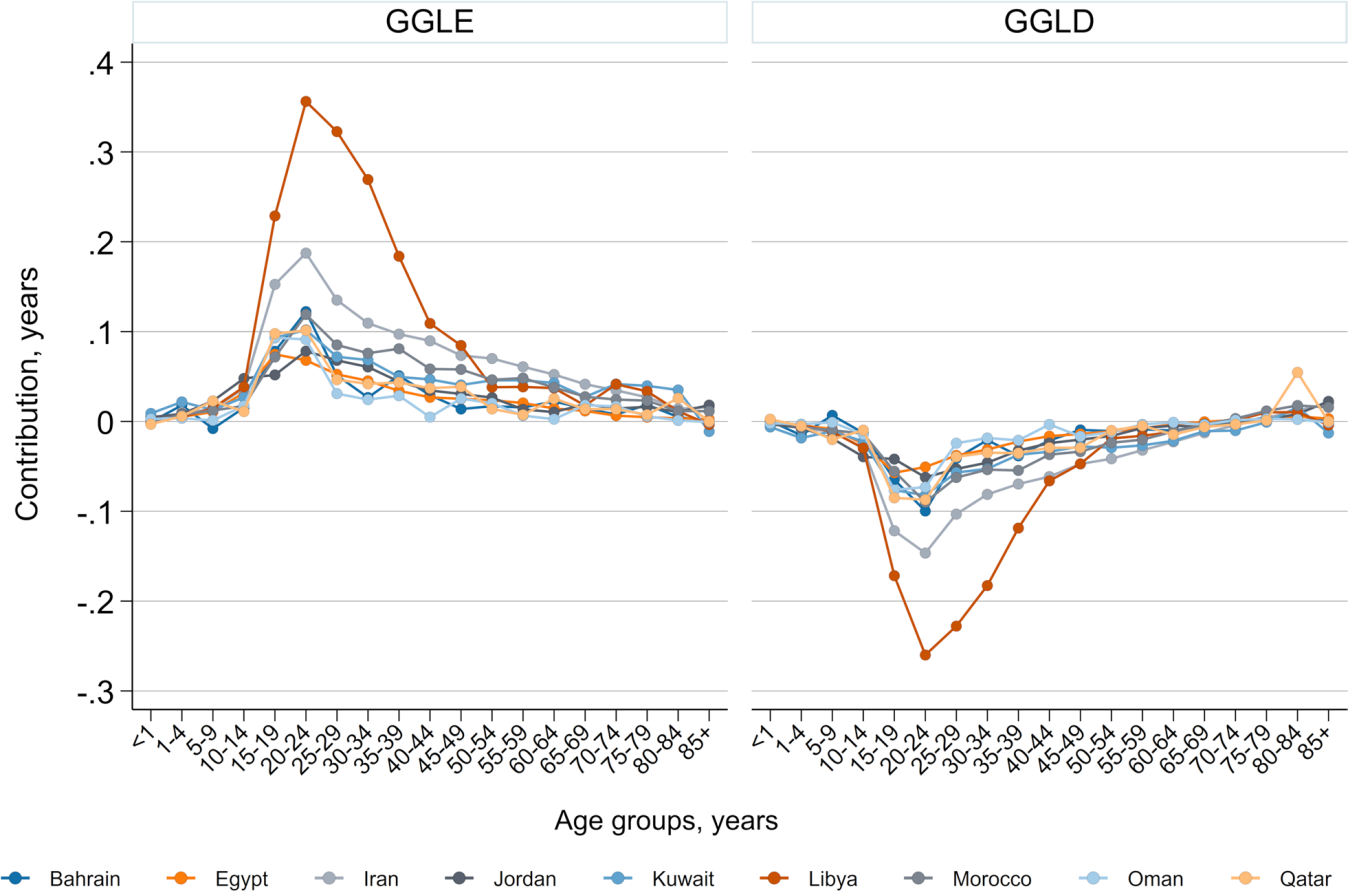
Age-specific contributions of injury deaths to the gender gap life expectancy (GGLE) and lifespan disparity (GGLD) across Eastern Mediterranean Region countries

Higher injury mortality rates in men than women in age group 20–24 years had the greatest contributions into GGLE due to injury death in all countries but Egypt and Oman, where age group 15–19 years had the greatest contributions to GGLE due to injury deaths (Fig. 1). Injury deaths in age groups 15–39 years accounted for 48% (0.4 years out of 0.8 years in Kuwait) to 74.5% (1.4 years out of 1.8 years in Libya) of the total GGLE attributable to injury. Similar patterns were seen for the GGLD with age-specific contributions of injury deaths to the total GGLD attributable to injuries generally peaked at age group 20–24 years (Fig. 1). Moreover, the relative contributions of age groups 15–39 years to the total injury attributable GGLD was even greater, ranging from 58.6% in Kuwait to 83.5% in Libya. All countries showed more than 70% (most countries over 80%) of total GGLE/GGLD in age groups under 55 years. Further detailed results can be found in Additional file 1.

### Cause specific contributions to the GGLE and GGLD

**Fig. 2:**
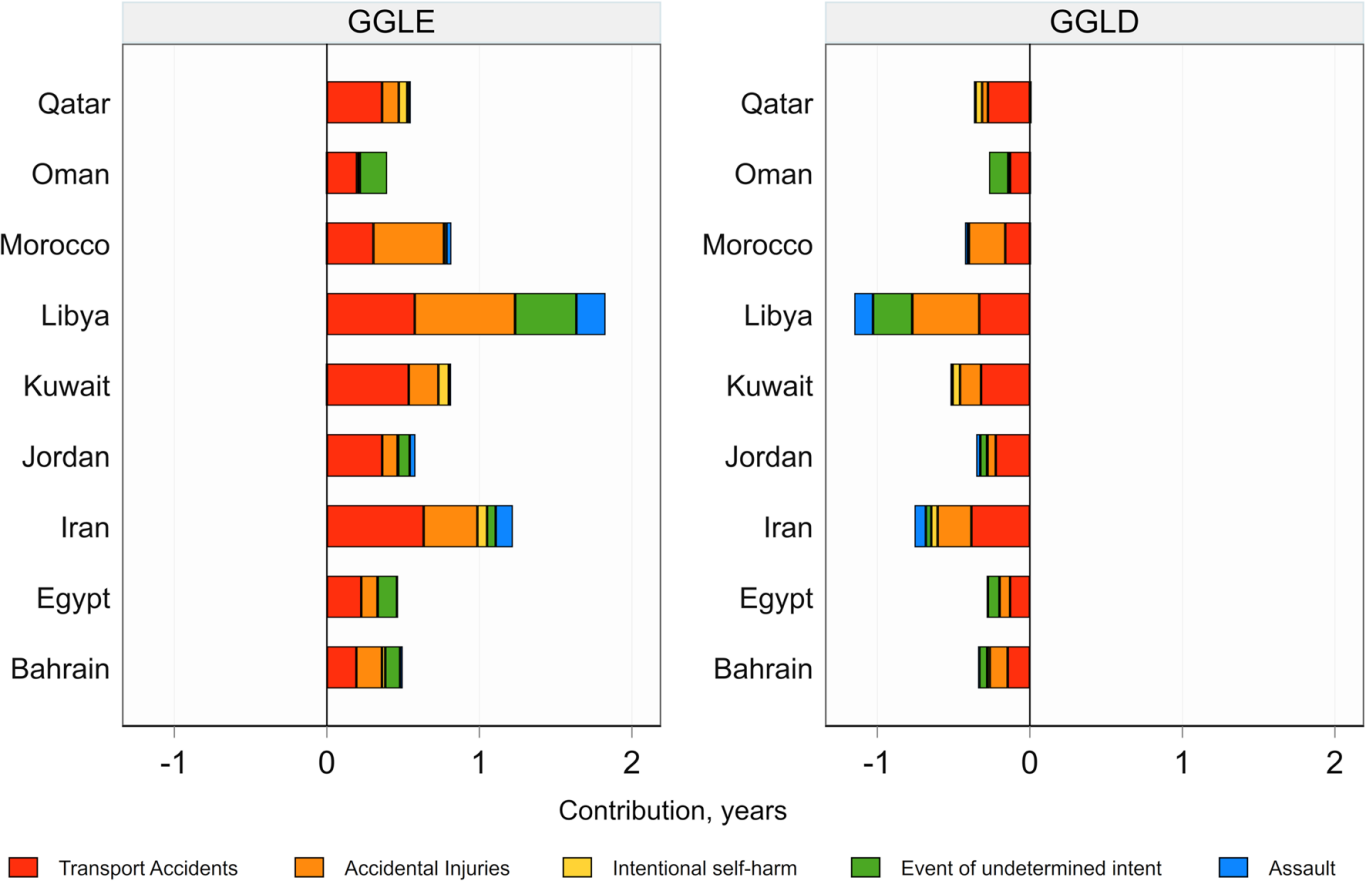
Cause-specific contributions of injury deaths to the gender gap in life expectancy (GGLE) and lifespan disparity (GGLD) across Eastern Mediterranean Region countries

Among the categories of injury-related deaths, transport accidents were the leading cause of the total GGLE/GGLD attributable to injury deaths in all countries but Libya and Morocco, where accidental injuries were the leading cause (Fig. 2). In absolute terms, the greatest contributions of transport accidents to both GGLE (0.6 years) and GGLD (− 0.4 years) were observed in Iran. On the other hand, the greatest relative contributions to GGLE and GGLD were reported in Kuwait (66.3%) and Qatar (77.2%), respectively. Following transport accidents, accidental injuries were generally the second important contributors to the total GGLE/GGLD attributable to injuries. Libya had the greatest absolute GGLE/GGLD for 3 out of 5 injury causes of deaths (i.e., accidental injuries, event of undetermined intent and assault). The greatest GGLE/GGLD due to intentional self-harm was reported in Kuwait (0.07/− 0.05 years). On contrary to the general pattern, mortality rates for event of undetermined intent in Kuwait and Morocco and for assault in Oman were higher in women compared with men, even though their contributions were negligible.

### Age- and cause-specific contributions of injury deaths to the GGLE and GGLD

In < 1-year age group, accidental injuries and event of undetermined intent were each the leading cause of the injury related GGLE/GGLD in four countries, with transport accidents being the leading cause in Oman (Additional file 1). In those aged 1–9 years, accidental injuries were the leading cause in Iran, Egypt, Bahrain and Morocco, while transport accidents were the leading contributor in other countries. In people aged 10 + years, transport accidents were generally the leading cause of the injury attributable to GGLE/GGLD with two main exceptions: Morocco where accidental injuries were the leading cause across all age groups, and Libya where accidental injuries were the leading cause in those aged 20–39 years accounting for around 25% of the total injury attributable GGLE/GGLD in these age groups. Although the age-cause contribution to the gender gap showed different results within each country, the highest rate of injury deaths was generally observed in men.

## Discussion

While the impact of injury deaths on LE and LD has been studied before (Fei et al. 2018; Fenelon et al. 2016), the contribution of injury deaths to the gender gap in LE and LD in EMR countries is not well understood. This study examined the contributions of injury deaths to the GGLE and GGLD in nine EMR countries. Our finding revealed that in most countries, injury deaths had a major contribution to both GGLE and GGLD in favor of women. In particular, compared to the other age-sex groups, men aged 15–24 years were the most vulnerable group affected by injury deaths, specifically transport accidents. There were cross-country variations in the age- and cause-specific contributions of injury death to the GGLD and GGLE. For instance, while transport accidents were the leading cause of the GGLE/GGLD attributable to injury in most countries, accidental injuries had the greatest contributions in Libya and Morocco.

Our study highlighted that injury deaths are among the major contributors of years of life lost in the EMR countries, which is in line with the global burden of disease studies (Mokdad et al. 2018; Khalil et al. 2018). According to the World Health Statistics, while the global mortality rate due to injuries remained stable or even declined for certain types of injuries (homicide, traffic accidents, suicide, and unintentional poisoning) over the past two decades (2000–2019), EMR countries experienced a consistent rise in injury-related mortality during this period (World Health Organization 2021). This can be explained partly by the long-lasting conflicts in some of the countries of the region such as Libya in our sample. The deaths due to the collective violence are mainly caused by conflict. The impact of war and conflict is not limited only to violence and assaults, in some studies there have been correlation between conflict and other intentional injuries i.e., suicide and self-harm (Murray et al. 2002; Mokdad et al. 2016; Rezaeian and Khan 2020). These countries had a dramatic rise in the number of intentional injuries including self-harm, interpersonal violence, collective violence and legal intervention from 1990 towards 2015. The number of deaths due to injuries had an upward trend with a surge between 2010 and 2015 (Mokdad et al. 2018).

The focus of this study was to investigate the impact of injury deaths on GGLE and GGLD, and our results clearly confirmed that injury deaths contributed to the gap between men and women in both LE and LD. There are not many studies that address this issue specifically in the EMR countries. The result of a study in the UK showed that across avoidable causes of death, injuries had second greatest contributions to the GGLE (Allel et al. 2021). In Sweden, the contribution of accidental injury to the GGLE more than doubled between 1997 and 2018 (Kiadaliri 2021). A study conducted in the United States confirms that although there is no particular gender pattern in deaths caused by injury, gender disparities tend to be consistent and persistent in mortality due to this cause (Sorenson 2011). There is no solid evidence that justify higher rates of mortality due to injury accidents in men compared to women because of physiological differences. Nevertheless, some studies confirm that socio-behavioral characteristics of men expose them more to mortality and disability caused by injuries (Rogers et al. 2010; Sorenson 2011). In general, men are more prone to risky behaviors such as use of alcohol and aggression or violence, high risk sports or driving style which lead to more injuries (Good et al. 2008; Smiler 2004). It should be highlighted that alcohol-related injuries in this region are significantly lower than other regions because of religious reasons. The consumption of alcohol in the EMR countries is one tenth of global consumption (World Health Organization, Regional Office for the Eastern Mediterranean 2022). Another factor that might have contributed to the higher rate of death from injuries in men in this region is the traditional gender prototypes, imposing the economic responsibility of the family on men as sole breadwinner and women taking care of the family at home (World Health Organization 2007). In other words, the variation in the roles of men and women in the society may increase the possibility of men working in high-risk jobs which, in turn, result in more injuries among them. Although this pattern is gradually changing, still it is the common frame in most countries of this region.

Transport accidents generally had the highest rank among all the injury death categories contributing to GGLE and GGLD. The burden of transport accident deaths is disproportionately borne by men and mainly among those aged between 15 and 25 years. This result is consistent with the other studies conducted in EMR countries (Yousefifard et al. 2021; Arafa et al. 2019; Tarlochan et al. 2022; Mbarek Sidi Mohamed Ben et al. 2020; Ghadi et al. 2018). Transport accidents are a common issue in all countries of the region with different income levels. The mortality rate caused by transport accidents in high-income countries in the region is three-fold higher than the average rate in the same income level countries worldwide (World Health Organization 2018). This means that the road traffic safety system and culture has not been developed as much as the economic growth and motorization in these countries. Similar to all injuries, mortality from transport accidents is not the same for men and women. This can be explained by the fact that men are more prone to take risk than women (e.g., driving faster). Another potential reason contributing to this gender gap is that due to different cultural and legislation reasons, women in the EMR countries drive less than their counterparts in other regions. Approximately 80% of the fatalities due to the transport accidents in this region happens in men, with Qatar and Bahrain having the highest mortality rate in men caused by transport accidents (93% and 91%, respectively) (World Health Organization 2010). Iran in particular, had the highest rate of death due to transport accidents. Although Iran indicated a considerable growth in LE during the last 4 decades due to marked reduction in mortality rate (Ebrahimi et al. 2020), when it comes to reduction in mortality due to transport accidents, it is still lagging far behind the other EMR countries and this calls to an urgent action for the prevention of transport accidents (Khorasani-Zavareh et al. 2009; Allel et al. 2022).

Young men aged between 15 and 24 years are at the highest risk of mortality due to injuries, specifically transport injuries. Since this age group is the main part of the economically productive age group of the society, higher mortality of this age group leads to greater productivity loss (World Health Organization 2018) and it can substantially contribute to greater LD. As indicated previously, LE may improve by reduction in mortality at any age while LD improves by saving more lives at younger ages. Recent improvements in healthcare services have led to a greater impact on the reduction of mortality at older age and consequently higher LE. The majority of countries in this study are losing productive years of their population due to injury deaths.

Our study highlighted that, injuries as one of the avoidable causes of death, significantly contribute to GGLE/GGLD, thus, implementing preventive strategies to reduce the deaths imposed by injuries could have a great impact on narrowing GGLE/GGLD. This is consistent with the finding of a study in Sweden that showed reduction in avoidable causes led to a greater LE gain in men and subsequently narrowing the GGLE with underlying role of inter-sectoral public health policies (Kiadaliri 2021). It is also argued that reduction in mortality rate can cause higher gain in LE in men because of more dispersed age distribution of death in men. This explains how the same mortality rate reduction in both men and women, results in narrowing the GGLE and also GGLD (Glei and Horiuchi 2007).

We acknowledge some limitations of our study. First, our results might not be generalizable to all the EMR as we included only 9 out of 22 countries in this study due to data availability. Even though these countries are in the same region, there are substantial variations in socioeconomic characteristics of each country that make the cross-country comparison challenging. Second, the WHO mortality database includes medically certified deaths as reported by the member countries. These data sources are known to suffer from coding errors, diagnostic inaccuracy, and underreporting. This can bias the estimates reported in the present study, albeit if the patterns of these errors and misreporting vary by gender. This is because we investigated GGLE/GGLD within each country and similar patterns across sexes would cancel out their effects on our estimates. Finally, following the current literature, we have used term “gender gap” in this study. However, it should be noted that what we used is birth sex (female, male) which differ from gender as a social construct.

## Conclusions

High injury deaths, particularly deaths due to the transport accidents, among young men had considerable contribution to the GGLE and GGLD among the EMR countries included in this study. These findings highlight the necessity to implement preventive policies to reduce the number of deaths caused by injuries. It is essential to consider the characteristics of the population most at risk of injury-related mortality when developing and implementing effective strategies. This study highlighted that premature death in young men caused by different types of injuries impose a great burden on countries. Thus, implementing preventive measures and targeting this high-risk group could potentially save a significant number of years of life lost due to injury-related mortality. It is important to highlight the magnitude of burden of injury deaths for policy makers and to emphasize that deaths caused by injuries are avoidable to a significant degree. Designing an injury surveillance program and embedding an intersectoral approach to prevent injury deaths will assist countries to unload the burden caused by injury deaths.

## Supplementary Information

**Additional file 1.** **Table A1.** Age- and cause-specific contributions to gender gap in life expectancy/life disparity in Bahrain. **Table A2.** Age- and cause-specific contributions to gender gap in life expectancy/life disparity in Egypt. **Table A3.** Age- and cause-specific contributions to gender gap in life expectancy/life disparity in Iran.** Table A4.** Age- and cause-specific contributions to gender gap in life expectancy/life disparity in Jordan.** Table A5.** Age- and cause-specific contributions to gender gap in life expectancy/life disparity in Kuwait.** Table A6.** Age- and cause-specific contributions to gender gap in life expectancy/life disparity in Libya.** Table A7.** Age- and cause-specific contributions to gender gap in life expectancy/life disparity in Morocco.** Table A8.** Age- and cause-specific contributions to gender gap in life expectancy/life disparity in Oman.** Table A9.** Age- and cause-specific contributions to gender gap in life expectancy/life disparity in Qatar.

## Data Availability

All data analysed in this study is shared publicly at the WHO website.

## Abbreviations

LE: Life expectancy
LD: Life disparity
GGLE: Gender gap in life expectancy
GGLD: Gender gap in life disparity
EMR: Eastern Mediterranean Region
WHO: World Health Organization
CVRS: Civil vital registration system
